# Osteogenesis imperfecta in a male holstein calf associated with a possible oligogenic origin

**DOI:** 10.1080/01652176.2020.1721611

**Published:** 2020-02-10

**Authors:** Xuying Zhang, Marc Hirschfeld, Julia Beck, Alexandra Kupke, Kernt Köhler, Ekkehard Schütz, Bertram Brenig

**Affiliations:** a Institute of Veterinary Medicine, University of Goettingen, Göttingen, Germany; b Department of Obstetrics and Gynecology, University Medical Center Freiburg, Freiburg, Germany; c Chronix Biomedical, Institute of Veterinary Medicine, Göttingen, Germany; d Institute of Virology, Philipps University Marburg, Marburg, Germany; e Institute of Veterinary Pathology, Justus Liebig University Gießen, Gießen, Germany

**Keywords:** Bovine, cattle, cow, osteodysplasia, osteodystrophy, osteogenesis imperfecta, brittle bone disease, congenital bone disorder, embryo transfer, malformation

## Abstract

**Background:**

Neuromusculoskeletal anomalies generally in combination with severe clinical symptoms, comprise a heterogeneous group of fairly common and mostly fatal disorders in man and animals. Osteogenesis imperfecta (OI), also known as brittle bone disease, causes bone fragility and deformity. Prominent extra-skeletal accessory manifestations of OI comprise blue/gray sclerae, hearing impairment, lung abnormalities and hypercalciuria. Cases of OI in cattle have been reported. However, no causative mutations have been identified in cattle so far.

**Aim:**

To report a possible oligogenic origin identified in a calf from clinically healthy parents suffering from OI.

**Materials and Methods:**

A neonatal embryo transfer male Holstein calf developing multiple fractures with bone tissue showing marked osteopenia was used for whole genome re-sequencing as well as its parents. In addition, 2,612 randomly chosen healthy Holstein cattle were genotyped as well as controls.

**Results:**

Sixteen candidate genes with potential protein-altering variants were selected revealing non-synonymous variants only within *IFITM5* and *CRTAP* genes. However, in-depth gene analysis did not result in the identification of a single causative mutation in the OI calf.

**Conclusion:**

The analysis of the OI case revealed a possible oligogenic origin of the disease attributable to additive effects of three candidate genes, i.e., *ABCA13*, *QRFPR*, and *IFTIM5*.

**Clinical relevance:**

Most OI cases in humans and domestic animals reported so far are caused by distinct dominant or recessive monogenic mutations, therefore a potential oligogenic additive genetic effect is a novel finding. Furthermore, the case presented here demonstrates that cross-species genetic analyses might not always be straightforward.

## 1. Introduction

Development and maintenance of the skeletal system requires complex processes, which are characterized by continuous modelling and re-modelling providing structural and reservoir functions for the body throughout life. Hence, it seems comprehensible that a variety of pathological conditions interfere with a physiological development of the skeleton including genetic defects, nutritional deficiencies and hormonal disorders. This prompted a group of researchers in the early 1970s to establish a nosology and classification of human skeletal disorders in order to facilitate definitive diagnosis and help to delineate variant or newly recognized conditions (Warman et al. 2011). Currently, this system includes 461 genetic disorders of the skeleton classified in 42 groups involving 437 genes (Mortier et al. 2019). Osteogenesis imperfecta (OI) together with other decreased bone density disorders form group 25 in this classification (Mortier et al. 2019).

OI, also known as brittle bone disease, affects about 1/10,000 – 1/20,000 live births in humans (Forlino and Marini 2016; Folkestad et al. 2017). OI patients exhibit prominent skeletal abnormalities causing bone fragility and deformity, with widely varying clinical severity. In addition, joint laxity, scoliosis, kyphosis, dentinogenesis imperfecta as well as craniofacial abnormalities may represent concomitant phenomena in OI patients. Prominent extra-skeletal accessory manifestations of OI comprise blue/gray sclerae, hearing impairment, lung abnormalities and hypercalciuria (Sillence et al. 1979; Rauch and Glorieux 2004; Shaker et al. 2015; Forlino and Marini 2016).

In humans, more than 90% of OI cases are caused by autosomal dominant mutations in the genes encoding type I collagen (*COL1A1*, collagen type I alpha 1 chain; *COL1A2*, collagen type I alpha 2 chain) (Rauch and Glorieux 2004). A minority of OI cases occur as a result of recessive mutations in various genes, leading to different functional defects from structural to enzymatic as well as from intracellular transport to chaperones (Forlino and Marini 2016). Most of these genes are involved in collagen metabolism, *e.g., BMP1* (bone morphogenetic protein 1), *CRTAP* (cartilage associated protein), *PLOD2* (procollagen-lysine, 2-oxoglutarate 5-dioxygenase 2), *FKBP65* (65-kDa FK506-binding protein), *HSP47* (heat shock protein 47), *IFITM5* (interferon induced transmembrane protein 5), *TRIC-B* (trimeric intracellular cation channel subtype B), *WNT1* (Wnt family member 1) and *SP7/OSX* (SP7 transcription factor/osterix) (Koide et al. 2006; Baek et al. 2010; Martinez-Glez et al. 2012; Semler et al. 2012; Laine et al. 2013; Farber et al. 2014; Guillen-Navarro et al. 2014; Gjaltema et al. 2016; Zhao et al. 2016). Depending on the causative gene or mutation, 13 different types of autosomal recessive and dominant OI are currently described in humans that are classified into five different groups based on their phenotypes (Thomas and DiMeglio 2016).

Cases of OI in Holstein cattle have been reported since 1983 (Denholm and Cole 1983; Lenffer et al., 2006)). However, no causative mutations have been identified in Holstein cattle so far (Denholm and Cole 1983; Agerholm et al. 1994). A study with focus on non-collagenous proteins in OI affected Texan Holstein cattle showed a severe depletion of osteonectin and bone proteoglycan in bones and phosphophoryn in teeth (Termine et al. 1984). OI has also been described in Charolais cattle (OMIA 000754-9913), sheep (OMIA 000754-9940), cats (OMIA 000754-9685) and dogs (OMIA 002112-9615, 000754-9615, 002126-9615, 001483-9615) (Omar 1961; Jensen et al. 1976; Cohn and Meuten 1990; Arthur et al. 1992; Evason et al. 2007; Lenffer et al., 2006)). However, only two forms of canine OI have been elucidated on a molecular level so far (OMIA 002112-9615, 001483-9615) (Campbell et al. 2000; 2001; Drogemuller et al. 2009; Schutz et al. 2013; Lenffer et al., 2006)) and only recently *COL1A1 de novo* mutations have been described in Fleckvieh and Red Angus (OMIA 002127-9913) (Bourneuf et al. 2017; Lenffer et al., 2006; Petersen et al. 2019).

The aim of the current study is to report a possible oligogenic origin identified in a Holstein calf suffering from OI.

## 2. Material and methods

### 2.1. Ethical statement

EDTA blood samples were collected during routine diagnostic parentage control with written owner consent. Blood samples were drawn exclusively by local veterinarians. The collection of samples was approved by the Lower Saxony State Office for Consumer Protection and Food Safety (33.19-42502-05-17A196) according to §8a Abs. 1 Nr. 2 of the German Animal Protection Law.

### 2.2. Case description of the calf suffering from OI

**Figure 1.**
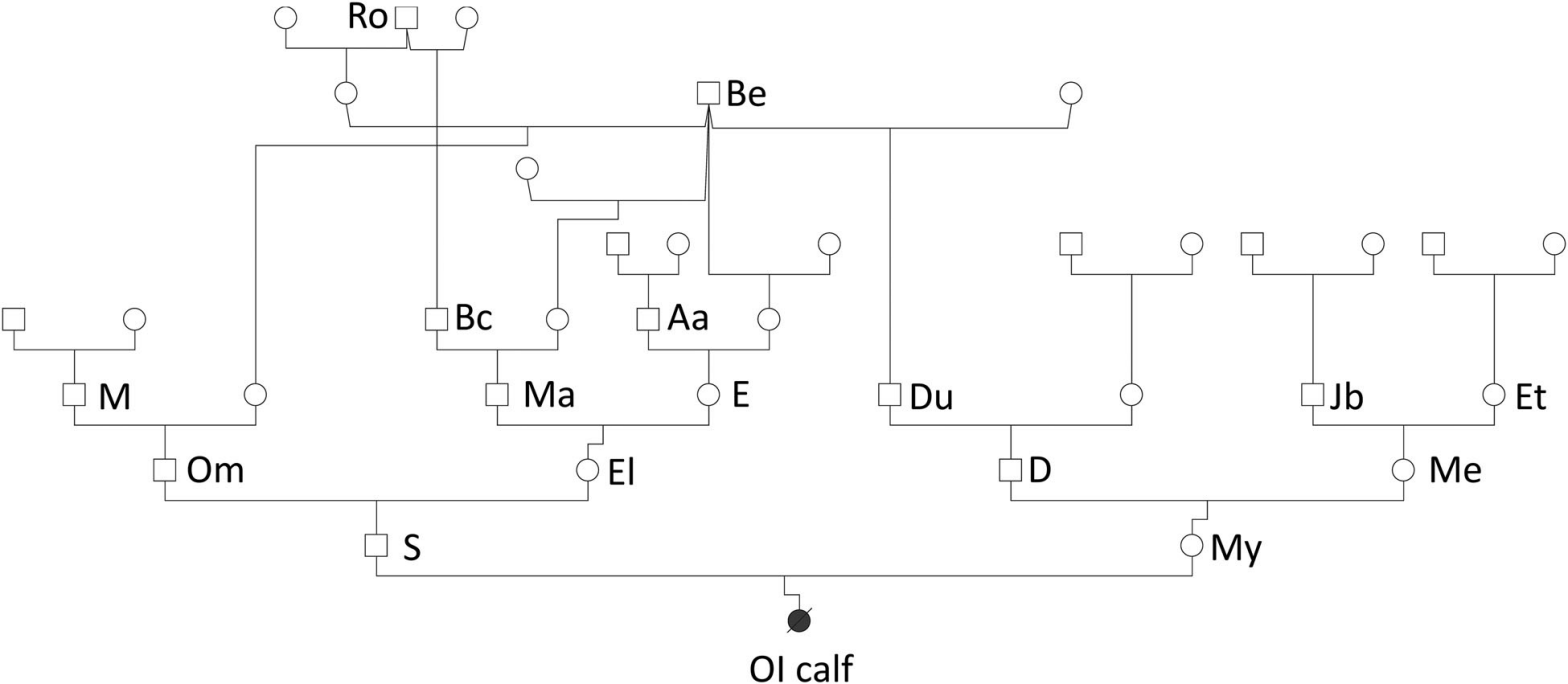
Pedigree of a male calf suffering from osteogenesis imperfecta. The pedigree depicts the six generations family structure of the affected calf. Selected individuals are indicated with letters. The pedigree was generated using the java webstart application Pedigree Chart Designer v2.0 (PCD).

The affected OI calf was bred by embryo transfer and delivered four weeks prematurely. There were no reports of a previous transmission or case of OI for both parents in their ancestry making a dominant trait unlikely. As several sires in the pedigree have been widely used in Holstein breeding a recessive monogenic inheritance was also unlikely, however was not excluded completely. As shown in Figure 1 the pedigree reflects a normal Holstein cattle family structure with no unexpected increased relatedness among animals due to inbreeding. It is evident that sires Ro and especially Be appear more often in the paternal as well as maternal ancestral line. While the father (S) of the OI calf has been used relatively often in artificial insemination with approximately 4.500 offspring, the mother (My) had only 12 offspring. S and My were mated only once using embryo transfer resulting in a twin birth with one male (OI calf) and one stillbirth female offspring.

Due to suckling weakness, bronchopneumonia and hypogammaglobulinemia, the OI calf was hospitalized shortly after birth. Four days after birth, the OI calf developed a comminuted fracture of the right tibia distal of the knee joint and two days later a similar fracture on the left side without any obvious traumatic influence. The OI calf had to be euthanized and was necropsied.

**Figure 2.**
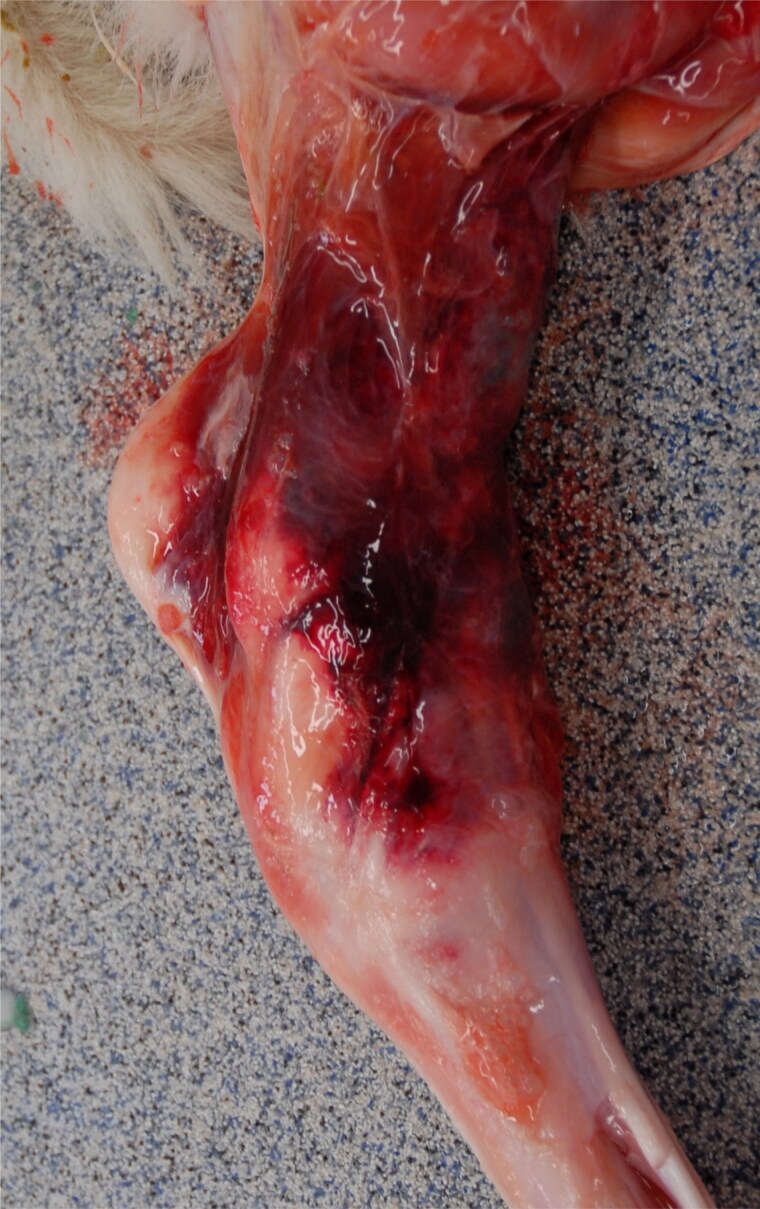
Gross lesions associated with a tibial fracture in male calf suffering from osteogenesis imperfecta. The picture shows periarticular hemorrhage in the hock region (medial view of the left hind limb) due to a comminuted fracture of the distal tibia (not shown).

**Figure 3.**
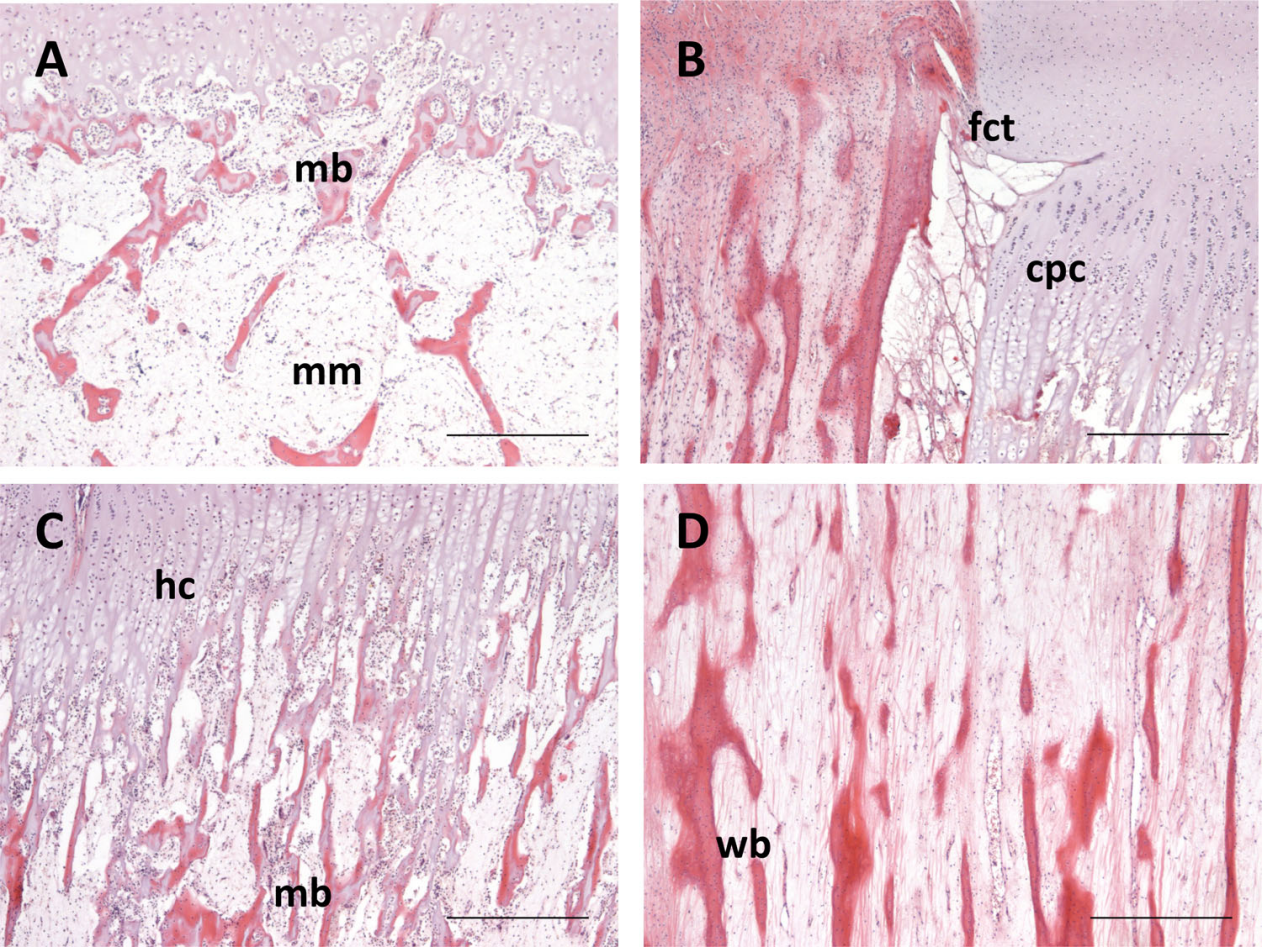
Bone photomicrographs of a male calf suffering from osteogenesis imperfecta. (A) Affected calf showing marked osteopenia, mucinous matter (mm) replacing bone marrow, (B) direct transition of columnar proliferative cartilage (cpc) to fibrous connective tissue (fct), (C) not clearly separated metaphyseal bone (mb) from the hypertrophic chondrocytes (hc) and (D) woven bone (wb) instead of lamellar bone; scale bar: 500 µm.

Post-mortem examination revealed comminuted fractures of both tibias approximately 2-5 mm distal of the epiphyseal plate (Figure 2). Fractures were accompanied by hemorrhages in the adjacent tissue. Additional fractures and disruptions of the articular cavities of the scapulae were determined. Other organs were unremarkable. Microscopic investigations revealed that the bone tissue showed marked osteopenia characterized by thin cortical bone and thin trabeculae of the spongiosa, often consisting of woven instead of lamellar bone. Only few osteoclasts were present throughout the sections. Multifocally, trabeculae were widely separated by abundant mucinous matter, replacing bone marrow cells. In some regions, a direct transition between columnar cartilage and fibrous connective tissue was observed (Figure 3 A-D). From the post mortem and histological examinations an OI was diagnosed.

### 2.3. Tissue preparation and histology

Tissues from the OI calf were fixed in 10% formalin. Thin bone sections were prepared with a diamond band saw and decalcified in EDTA at 37 °C for up to 14 days, rinsed in tap water over night and routinely processed and embedded in paraffin. Sections of 4 µm thickness were dewaxed in xylene, rehydrated in a decreasing ethanol series and H&E stained in an autostainer (Microm, HMS 740, Dreieich, Germany).

### 2.4. DNA isolation and Sanger sequencing

DNA isolation from blood, liver tissue (OI calf) and semen samples was performed using MagNa Pure LC DNA Isolation Kit I (Roche Diagnostics, Mannheim, Germany) or a modified salting out procedure (Miller et al. 1988).

Using NCBI/Primer-BLAST, PCR primer pairs were designed (supplementary material Table 1) (Ye et al. 2012). A total volume of 25 μl was prepared for each PCR reaction, including 1× PCR reaction buffer plus 20 mM MgCl_2_, 0.5 μl of 10 mM dNTPs, 1 U FastStart Taq Polymerase (Roche), 1 μl of 10 pmol/μl each primer (Sigma-Aldrich, Hamburg, Germany), and 1 μl of 20 ng/μl DNA. Reaction conditions included 95 °C for 10 min, then 30 cycles of 95 °C for 30 s, primer-specific annealing temperatures (supplementary material Table 1) for 30 s and 72 °C for 45 s, followed by final elongation at 72 °C for 7 min.

MOVE TABLE 1 to paragraph 3.1.

**Table 1.** Detection of DNA variants in osteogenesis imperfecta candidate genes.

			Genotypes (OI-family)				Controls		
Gene	Position	Variant	OI calf	My^a)^	S^b)^		Ref/Ref	Ref/Alt	Alt/Alt
*IFITM5*	11:g.82966108	rs209568970	Alt/Alt	Ref/Alt		Ref/Alt	1	4	1
*CRTAP*	22:g.7459319	rs466604499	Ref/Alt^c)^	Ref/Alt		Ref/Ref	1	1	0

^a)^My: Mother (see also Figure 1); ^b)^S: Father (see also Figure 1); ^c)^Ref: Reference allele, Alt: Alternative allele.

Purification of PCR products was performed using Rapid PCR Cleanup Enzyme Set (New England Biolabs GmbH, Frankfurt am Main, Germany). Amplicons were sequenced using the BigDye^TM^ Terminator v3.1 Cycle Sequencing Kit (Applied Biosystems, Thermo Fisher Scientific GmbH, Schwerte, Germany) and fragments were separated on an ABI PRISM 3130xl Genetic Analyzer (Life Technologies, Darmstadt, Germany) following the manufacturers´ protocols. DNA sequences were aligned using SeqMan Pro software (DNASTAR Lasergene, Madison, WI, USA).

### 2.5. Next generation sequencing and data analysis

Paired-end sequencing was performed using the TG NextSeq 500/550 High Output Kit v2 (Illumina, München, Germany) on a NextSeq500 platform. Sequence arithmetics (average depth of coverage, per base coverage) were calculated using bed- and samtools (Li et al. 2009; Quinlan and Hall 2010; Quinlan 2014). For the OI affected calf, its mother (My) and father (S) an average coverage of 36.3x, 27.2x and 38.3x was obtained, respectively. Details of sequencing read depth and average coverage in regions of known OI causative genes are shown in supplementary material Figure 1. Sequences were aligned either to bovine genome build UMD3.1 or ARS-UCD1.2 using BWA-MEM with alignment parameters t = 8, A = 1, B = 4, O = 6, E = 1 and k = 31. For variant calling sorted bam-files were converted to vcf using samtools and bcftools (Li et al. 2009). Vcfs were imported into SNP & Variation Suite 8.8.3 (GoldenHelix, Bozeman, MT, USA) for calling of SNPs and indels. Sequences were aligned to 3,093 samples of Run 7 of the 1000 bulls genome project (Hayes and Daetwyler 2019). SVDetect and delly was used for identification of larger genomic structural variations (Zeitouni et al. 2010; Rausch et al. 2012). CANDID v1.1 and GUILDify v2.0 were used for gene prioritization (Hutz et al. 2008; Tranchevent et al. 2011; Aguirre-Plans et al. 2019). Prediction of functional impact of variants was done using SIFT (Vaser et al. 2016).

### 2.6. Genotyping of variants

Unless otherwise specified all positions refer to bovine genome assembly ARS-UCD1.2. Genotyping of *ABCA13* variants*, i.e.,* rs381405831 (4:g.7398705T > C) and rs110593220 (4:g.7324346T > C) was realized by multiplex-fluorescence resonance energy transfer (FRET)-PCR (Forster 1948). ABCA13_rs381405831 primers and ABCA13_rs110593220 primers (supplementary material Table 1) were designed using NCBI/Primer-BLAST (Ye et al. 2012). Probe/anchor, rs381405831_6-FAM/ROX and rs110593220_6-FAM/Cyanine 5 (supplementary material Table 1), were designed using MeltCalc (Schutz and von Ahsen 1999; von Ahsen et al. 1999). Multiplex PCR was performed on LightCycler 480 (Roche) in a total volume of 15 μl using FastStart Taq DNA Polymerase, dNTPack (Roche). One reaction mix included 0.75 U Faststart Taq DNA Polymerase, 3 nmol dNTPs, 6 pmol of each primer and probe, 1x Q-solution (Qiagen, Hilden, Germany), 1x PCR reaction buffer plus 20 mM MgCl_2_, and 20 ng of DNA. Cycling conditions were 95 °C for 10 min, followed by 35 cycles of 95 °C for 30 s, 61 °C for 30 s and 72 °C for 30 s. The final elongation step was 72 °C for 7 min. Melting analysis was performed using the appropriate filter set (483–610 filter comb for rs381405831; 483–670 filter comb for rs110593220) and the following program: 95 °C for 30 s, 37 °C for 30 s, 95 °C continuous acquisition mode (2 acqui./°C), ramp rate 0.14 °C/s, followed by 37 °C for 10 s.

Amplification refractory mutation system (ARMS) (Newton et al. 1989) was designed to genotype *QRFPR* variant rs209556962 (6:g.4198178C > T). Primers, rs209556962_WTfwd/rev and rs209556962_MUTfwd/rev (supplementary material Table 1), were designed using NCBI/Primer-BLAST (Ye et al. 2012), to amplify the wild type or mutant specific fragment with an internal amplification control sequence in a single reaction. The ARMS-PCR was performed on LightCycler 480 (Roche) in a total volume of 25 μl. One reaction mix included 1 U Faststart Taq DNA Polymerase (Roche), 5 nmol dNTPs (Roche), 10 pmol of each primer, 1x PCR reaction buffer plus 20 mM MgCl2 (Roche), 1x EvaGreen (Jena Bioscience, Sidney, Australia) and 10 ng of DNA. Cycling conditions were 95 °C for 10 min, followed by 28 cycles of 95 °C for 30 s, 66 °C for 30 s and 72 °C for 25 s. Final elongation step was 72 °C for 7 min. Melting analysis was performed using the appropriate filter set and the following program: 95 °C for 1 min, 40 °C for 1 min, 75 °C for 1 s, 90 °C continuous acquisition mode (25 acqui./°C), ramp rate 0.02 °C/s.

PCR-restriction fragment length polymorphism (PCR-RFLP) was used to genotype *IFITM5* rs209568970 (11:g.82966108C > A). The IFITM5_RFLP primers (supplementary material Table 1) were designed using NCBI/Primer-BLAST (Ye et al. 2012). Cycling conditions were 95 °C for 10 min, followed by 36 cycles of 95 °C for 30 s, 61 °C for 30 s and 72 °C for 15 s. Final elongation step was 72 °C for 7 min. Total volume of 25 μl RFLP master mix was prepared with 10 μl PCR product, 20 U *Ban*II (NEB), 1x CutSmart Buffer (NEB) and 1x EvaGreen (Jena Bioscience, Germany). On LightCycler 480 (Roche), digestion mixture was incubated at 37 °C for 4 h. Melting analysis was performed using the appropriate filter set and the following program: 70 °C for 1 s, 95 °C continuous acquisition mode (5 acqui./°C), ramp rate 0.11 °C/s, followed by 37 °C for 1 s. Nomenclature of variants was done according to the standard recommendations (Eilbeck et al. 2005; Richards et al. 2015; den Dunnen 2016; den Dunnen et al. 2016).

### 2.7. Statistical analysis

Hardy-Weinberg equilibrium was calculated as described (Rodriguez et al. 2009). χ^2^ scores were converted to *p*-values using RStudio (Team 2018).

### 2.8. Data availability

The data that support the findings of this study have been deposited with the European Nucleotide Archive ENA under project number PRJEB34325 (ERP117210). Individual accession numbers are: OI affected calf (ERX3676071), mother (My, ERX3676072) and father (S, ERX3676073).

## 3. Results

### 3.1. Analysis of known candidate genes causative for osteogenesis imperfecta

In a first attempt a comparative sequencing of all potential OI candidate genes reported in humans, *i.e., COL1A1*, *COL1A2*, *IFITM5*, *SERPINF1*, *CRTAP*, *LEPRE1*, *PPIB*, *SERPINH1*, *FKBP10*, *SP7*, *BMP1*, *TMEM38B* and *WNT1*, was performed in a cohort comprising the affected OI calf, its parents and 2,612 randomly chosen DNA of healthy Holstein cattle as controls. Only within *IFITM5* and *CRTAP* non-synonymous variants were detected (Table 1).

In *IFITM5* a homozygous missense variant (NC_037338.1:g.82966108C > A) was identified in the OI calf resulting in an amino acid exchange ENSBTAP00000041562.3:p.Ala30Ser. This variant was heterozygous in the parents and would therefore correspond to a recessive inheritance of the disorder. However, the homozygous alternative genotype was also detected in a healthy control excluding this variant as single causative candidate. Moreover, this variant seems to be tolerated as predicted by SIFT (0.42).

In *CRTAP* a heterozygous inframe 9 bp-deletion (NC_037349.1:g.7459319_7459327del) resulting in a truncation of three amino acids (ENSBTAP00000028588.4:p.Val18_Ala20del) was identified in the OI calf reflecting a dominant defect if causative for the disorder. The deletion was inherited by the mother and was also detected in one healthy control excluding this variant as causative.

The analysis of hitherto known candidate genes associated with human OI unexpectedly did not result in the identification of any single causative mutation in the OI calf. These results prompted us to perform a whole genome re-sequencing of the OI calf and its parents.

### 3.2. Next generation sequencing of OI calf and parents

5´-, 3´-UTRs, splice sites and coding regions of the OI calf were analyzed for the presence of recessive homozygous polymorphisms, *de novo* mutations, compound heterozygous polymorphisms and larger genomic structural variations. The basic work flow for NGS analysis is depicted in supplementary material Figure 2. No structural variations (supplementary material Tables 2–7), *de novo* mutations or compound heterozygous polymorphisms were identified qualifying as candidates. A total of 10,995,619 recessive homozygous variants were identified in the OI calf. 481 protein-altering variants (470 missense variants, 9 nonsense mutations, 2 frameshift variants) remained after filtering (supplementary material Table 8). To further narrow potential OI causative variants, gene prioritization was performed using osteogenesis, bone fracture, loose joint, brittle teeth, white sclera and bone deformity as keywords. This resulted in a list of 1,436 prioritized genes using GUILDify (supplementary material Table 9) and 1,038 prioritized genes using CANDID (supplementary material Table 10). The two candidate gene lists and 481 protein-altering variants were compared resulting in 38 candidate genes present in both lists (supplementary material Table 11). After prediction of functional impact of the filtered variants 16 genes remained as candidates (supplementary material Table 12), i.e., *QRFPR*, *ABCA13*, *MMAB*, *CCDC137*, *PAG21*, *KRT20*, *SOX8*, *GTPBP4*, *FAT4*, *SPOCK2*, *CACNA1I*, *COL6A6*, *AGER*, *MXRA5*, *CRTAC1* and *CALCR*. Validation of all detected protein-altering variants was performed in these 16 genes, including 2 stop gain variants, 2 start lost mutations, 2 splice sites variants, 16 missense mutations, 1 inframe deletion and 1 frameshift insertion. Furthermore, in 8 functionally unknown genes 7 stop gain variants and 1 frameshift insertion were analyzed. All variants were comparatively sequenced in the affected calf, its parents and healthy control cattle (supplementary material Table 12). None of the filtered and selected variants proved to be the only causative mutation.

**Table 2.** Iterative determination of genotype frequency of osteogenesis imperfecta causative variants in 2,612 random samples of Holstein cattle.^a^

Gene	Position	Variant	Ref > Alt	Ref_Ref	Ref_Alt	Alt_Alt
*ABCA13*	4:g7398705	rs381405831	T > C	1479	978	155
*ABCA13*	4:g7324346	rs110593220	T > C	1104	1442	66
*QRFPR*	6:g4198178	rs209556962	C > T	19	28	16
*IFITM5*	11:g82966108	rs209568970	C > A	6	10	0

^a^*ABCA13* genotypes were determined in 2,612 samples. Samples harboring the homozygous alternative alleles were genotyped for the stop gain variant in *QRFPR*. The remaining 16 samples harboring the homozygous *QRFPR* variant were genotyped for the *IFITM5* variant.

## 4. Discussion

Similar to humans, where OI is classified as orphan disease, OI also seems to be a rare disorder with low prevalence in domestic animals as estimated from the number of specific cases reported so far (Semler et al. 2019). Hence, it was a fortunate coincidence that such a rare case of an OI calf had been reported. In an initial attempt to identify causative mutations, all known OI associated genes that have been identified in humans were comparatively sequenced between the affected calf and its parents. Surprisingly, only two protein-altering variants in *IFITM5* and *CRTAP* were identified. From the direct comparative sequencing of known OI candidate genes it was evident that the defect of the calf must have been the result of other and/or additional mutations in so far unassociated genes. To determine which further loci and genes could be involved, a whole-genome re-sequencing of the OI calf and its parents was conducted and sequences examined regarding functional and structural variants. In combination with a gene prioritization it was finally possible to obtain a short list of 16 functional relevant candidate genes and eight additional loci with loss of function due to nonsense or frameshift mutations (supplementary material Table 12). All variants were genotyped in the OI family and unrelated healthy control cattle. Variants in *AGER*, *SOX8*, *PAG21* and two loci (ENSBTAG000000313, NSBTAG000000303) were excluded because the OI calf was either homozygous wild type or heterozygous. For the remaining variants, the OI calf carried the homozygous mutated genotype. Therefore, different combinations of the remaining variants were tested in respect to their occurrence in the cattle population (2,612 random samples). Finally, the variants in *ABCA13* (rs381405831, rs110593220), *QRFPR* (rs209556962) and *IFITM5* (rs209568970) together were uniquely present only in the OI calf (Table 2).

None of the randomly tested individuals harbored all three variants in homozygosity simultaneously. A comparable combination of three genetic variants causing a childhood-onset cardiomyopathy has been described recently in humans (Gifford et al. 2019). In this report, the transmission of three missense variants in three different genes from unaffected or asymptomatic parents to their children has been described. While in this example the three missense variants were present as heterozygous genotypes in affected individuals, in other studies combinations of two or more genes in homozygous individuals have been reported to influence the disease risk. The development of breast cancer in humans, for instance, seems to be depending on the number of high-risk alleles present in *CYP17* and *HSD17B1* (Feigelson et al. 2001). Women harboring homozygous high-risk alleles in these two genes show the highest relative risk to develop advanced breast cancer. In an earlier study, the presence of homozygous high-risk alleles in three genes, i.e., *CYP17*, *CYP1A1*, *COMT*, have been shown to influence the development of breast cancer (Huang et al. 1999). Similar findings were made with common variants affecting cardiovascular disease risk (Simonson et al. 2011). Up to 30% of the variation in the so-called Framingham Risk Score for cardiovascular disease risk could be explained by the combination of 10 highly associated SNPs. From these and several more studies it is evident that a number of genetic diseases might be caused by the concerted action of otherwise insignificant, uninformative or weakly predictive independent variants (Simonson et al. 2011).

*ABCA13* is a member of the ATP-binding cassette subfamily A and has a basic function as transmembrane transporter (Albrecht and Viturro 2007). *ABCA13* is highly expressed in bone marrow stromal cells which can differentiate into bone, cartilage, adipocytes and hematopoietic supporting tissue (Krebsbach et al. 1999). A highly significant deviation from HWE of the homozygous carriers of the alternative allele (rs110593220) was detected within the 2,612 samples with χ^2^ = 252.97 (p = 1.2e-55). This variant results in an amino acid exchange at position 4,307 (of 4,885) (ENSBTAP00000034167.5p:Gln4307Arg) or 4,393 (of 4971) (ENSBTAP00000053807.3:pGln4393Arg) of ABCA13 depending on the isoform. According to SIFT this alteration alone is expected to be tolerated although it is positioned within the conserved ABC2_membrane_3 protein domain of the ATP binding cassette family of proteins (Reizer et al. 1992; Vaser et al. 2016). For *QRFPR* a direct involvement in bone formation has already been demonstrated and *QRFPR*^-/-^ mice show thin osteochondral growth plates, thickened trabecular branches and reduced osteoclast numbers. As the stop gain variant in *QRFPR* had been included on the bovine BeadChip (DEU_QRFPR4152699_4152699, DEU_QRFPR4152699_4152699_r) it was possible to determine the frequency of the genotypes in a larger cohort of 139,364 cattle showing a significant under-representation of the affected homozygous genotype (χ^2^ = 9.7, p = 0.008). This variant results in a truncation of QRFPR at amino acid position 412 (of 431) and effects the cytoplasmic domain at the C-terminal end of the protein which is usually the binding site of G- and accessory proteins with G-protein coupled receptors to facilitate intracellular signaling (Latorraca et al. 2017).

The under-representation of both homozygous genotypes in *ABCA13* and *QRFPR* in the population already indicated a deleterious effect. Most OI cases in humans and domestic animals reported so far are caused by distinct dominant or recessive monogenic mutations, therefore a potential oligogenic additive genetic effect was a novel finding. On the other hand, it is noteworthy that although the accuracy and representation of the currently available bovine reference genome has increased significantly over the last years, still approximately 3% of the genome remains uncovered hitherto (ARS-UCD1.2) (Coordinators 2016; Hayes and Daetwyler 2019). Therefore, it cannot be completely excluded that further causative variants could exist in the OI calf in unassembled, unmapped or poorly characterized chromosomal regions. However, due to the average depth of sequencing coverage and read depth of the OI calf and its parents it can be estimated that approximately 95% of the individual variants should have been detected (Taylor et al. 2016). Another explanation for the development of the defect could be epigenetic effects together with altered gene expression as a result of the *in vitro* embryo reproductive technologies. Such disorders have been described in cattle as consequence of assisted reproductive technologies resulting in reduced developmental capacity of *in vitro* bred embryos (Urrego et al. 2014). Unfortunately, the liver tissue that was provided after euthanization and section of the calf had not been stored correctly for RNA extraction and hence this question could not be addressed appropriately using RNASeq for example. With this in mind, the most apparent non-excludable interpretation of the data presented here is that an additive effect of the variants in *ABCA13*, *QRFPR* together with *IFITM5* contributed to the development of OI.

## 5. Conclusions

The analysis of the OI case revealed a possible oligogenic origin of the disease attributable to additive effects of three candidate genes, i.e., *ABCA13*, *QRFPR*, and *IFTIM5*. All three genes are directly or indirectly involved in bone development and formation or have been described as causative for human OI previously. The elucidation of an additive genetic effect demonstrates the complexity of the disease. The pathogenesis of the OI case presented here may be unique, however, demonstrates that cross-species genetic analyses might not always be straightforward.
